# Prognostic Performance of Peripheral Blood Biomarkers in Identifying Seropositive Individuals at Risk of Developing Clinically Symptomatic Chagas Cardiomyopathy

**DOI:** 10.1128/spectrum.00364-21

**Published:** 2021-08-25

**Authors:** Subhadip Choudhuri, Suresh K. Bhavnani, Weibin Zhang, Valentina Botelli, Natalia Barrientos, Facundo Iñiguez, Maria Paola Zago, Nisha Jain Garg

**Affiliations:** a Department of Microbiology and Immunology, University of Texas Medical Branch, Galveston, Texas, USA; b Institute for Human Infections and Immunity, University of Texas Medical Branch, Galveston, Texas, USA; c Department Preventive Medicine and Community Health, University of Texas Medical Branch, Galveston, Texas, USA; d Servicio de Cardiología, Hospital San Bernardo, Salta, Argentina; e Instituto de Patología Experimental, CONICET-Universidad Nacional de Salta (UNSa), Salta, Argentina; Labcorp

**Keywords:** infectious disease, Chagas cardiomyopathy, *Trypanosoma cruzi*, biomarkers’ prognostic performance, predictive risk analysis

## Abstract

Biomarkers for prognosis-based detection of Trypanosoma cruzi-infected patients presenting no clinical symptoms to cardiac Chagas disease (CD) are not available. In this study, we examined the performance of seven biomarkers in prognosis and risk of symptomatic CD development. T. cruzi*-*infected patients clinically asymptomatic (C/A; *n* = 30) or clinically symptomatic (C/S; *n* = 30) for cardiac disease and humans who were noninfected and healthy (N/H; *n* = 24) were enrolled (1 − β = 80%, α = 0.05). Serum, plasma, and peripheral blood mononuclear cells (PBMCs) were analyzed for heterogeneous nuclear ribonucleoprotein A1 (hnRNPA1), vimentin, poly(ADP-ribose) polymerase (PARP1), 8-hydroxy-2-deoxyguanosine (8-OHdG), copeptin, endostatin, and myostatin biomarkers by enzyme-linked immunosorbent assay (ELISA) and Western blotting. Secreted hnRNPA1, vimentin, PARP1, 8-OHdG, copeptin, and endostatin were increased by 1.4- to 7.0-fold in CD subjects versus N/H subjects (*P* < 0.001) and showed excellent predictive value in identifying the occurrence of infection (area under the receiver operating characteristic [ROC] curve [AUC], 0.935 to 0.999). Of these, vimentin, 8-OHdG, and copeptin exhibited the best performance in prognosis of C/S (versus C/A) CD, determined by binary logistic regression analysis with the Cox and Snell test (*R*^2^_C&S_ = 0.492 to 0.688). A decline in myostatin and increase in hnRNPA1 also exhibited good predictive value in identifying C/S and C/A CD status, respectively. Furthermore, circulatory 8-OHdG (Wald χ^2^ = 15.065), vimentin (Wald χ^2^ = 14.587), and endostatin (Wald χ^2^ = 17.902) levels exhibited a strong association with changes in left ventricular ejection fraction and diastolic diameter (*P* = 0.001) and predicted the risk of cardiomyopathy development in CD patients. We have identified four biomarkers (vimentin, 8-OHdG, copeptin, and endostatin) that offer excellent value in prognosis and risk of symptomatic CD development. Decline in these four biomarkers and increase in hnRNPA1 would be useful in monitoring the efficacy of therapies and vaccines in halting CD.

**IMPORTANCE** There is a lack of validated biomarkers for diagnosis of T. cruzi-infected individuals at risk of developing heart disease. Of the seven potential biomarkers that were screened, vimentin, 8-OHdG, copeptin, and endostatin exhibited excellent performance in distinguishing the clinical severity of Chagas disease. A decline in these four biomarkers can also be used for monitoring the therapeutic responses of infected patients to established or newly developed drugs and vaccines and precisely inform the patients about their progress. These biomarkers can easily be screened using the readily available plasma/serum samples in the clinical setting by an ELISA that is inexpensive, fast, and requires low-tech resources at the facility, equipment, and personnel levels.

## INTRODUCTION

Trypanosoma cruzi causes Chagas disease (CD), which is endemic in the Americas. The prolonged burden of CD affects 8 to 10 million people, and an additional 70 million are exposed to risk of infection every year (1). Because of migration of T. cruzi*-*infected persons from areas where CD is endemic to areas of nonendemicity and transmission of infection by blood or organ donation and maternal-fetal routes, CD is a global health concern (2, 3).

Several assays, including serology and PCR tests detecting parasite-specific antibodies and DNA, respectively, and hemoculture or microscopic observation of blood parasites are employed for diagnosis of infection (4–7). Typical T. cruzi infection results in an acute parasitemic phase followed by lifelong low-level parasite persistence that contributes to chronic inflammation and oxidative stress (8). Consequently ∼30% of infected individuals develop Chagas cardiomyopathy. The current toolbox for scrutinizing infected individuals progressing from clinically asymptomatic (C/A) to clinically symptomatic (C/S) CD is limited.

Secreted host molecules have been evaluated to determine their diagnostic potential in chronic CD. For example, systemic levels of gamma interferon (IFN-γ) and other inflammatory cytokines were elevated in C/S patients, and those of interleukin-2 (IL-2) and IL-17 were increased in C/A CD patients (9, 10). Others noted elevated serum levels of IL-6 and C-reactive protein (CRP) in Chagas cardiomyopathy patients (11, 12). Genetic polymorphisms of the 3′ untranslated region (UTR) associated with increased IL-12B expression and the C-terminal region associated with low IL-10 expression were found to be dominantly present in Chagas cardiomyopathy patients (13, 14). Plasma levels of NT-proB-type natriuretic peptide (BNP) and angiotensin converting enzyme (ACE2) were elevated in CD patients with an abnormal electrocardiogram (15, 16). Global proteomic analysis of plasma and peripheral blood mononuclear cells (PBMCs) of T. cruzi-infected rodents and humans identified heterogeneous nuclear ribonucleoprotein A1 (hnRNPA1), gelsolin, myosin regulatory light chain-2 (MYL2), vimentin, and vinculin as potential biomarkers of CD (17–19).

T. cruzi-induced oxidative stress enhanced the cardiac and circulatory 8-hydroxy-2-deoxyguanosine (8-OHdG [a DNA damage biomarker]), poly(ADP-ribose) polymerase (PARP1 [a DNA repair enzyme]), and lipid peroxide (LPO) levels in CD-infected mice and humans (20, 21). PARP1 had a regulatory role in proinflammatory and profibrotic gene expression in CD (22, 23), and PARP1 depletion improved heart function in CD-infected mice (21). The release of 8-OHdG, PARP1, and LPO may provide potential biomarkers of CD progression.

In recent years, new biomarkers of cardiovascular dysfunction were developed. Copeptin (a 4.2-kDA glycopeptide) was useful in monitoring the severity of post-cardiac arrest syndrome (24). Endostatin (a 20-kDa collagen XVIII fragment, antiangiogenic) levels were increased in cardiovascular disorders (25, 26). Cardiac-specific deletion of myostatin (a transforming growth factor β [TGF-β] family member) reduced muscle atrophy (27), and myostatin-targeting therapies have been proposed for treatment of cachexia and muscular dystrophy. The clinical relevance of copeptin, endostatin and myostatin in CD is not known.

In this study, we measured seven potential biomarkers in serum, plasma, and PBMCs of T. cruzi-infected patients presenting a spectrum of CD symptoms. We evaluated the clinical relevance and prognostic value of these markers in identifying the risk of Chagas cardiomyopathy development. Our findings indicate that vimentin, 8-OHdG, copeptin, and endostatin would be useful in monitoring the risk of symptomatic CD progression and/or efficacy of current and newly developed drugs and vaccines in halting CD.

## RESULTS

### Prognostic performance of proteome identified biomarkers in CD.

**FIG 1:**
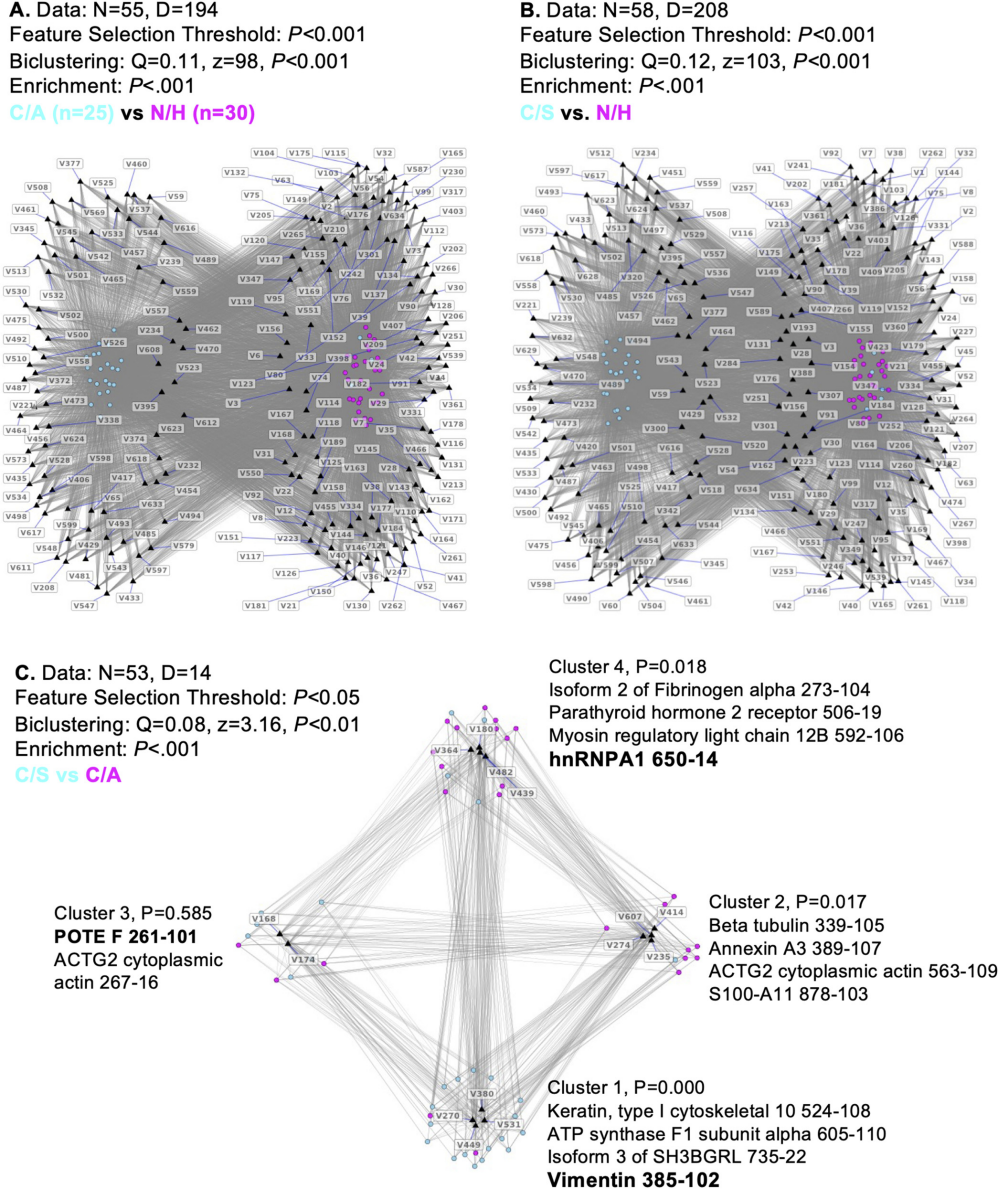
Bipartite analysis of the proteome data set for the identification of prognostic biomarkers of CD progression. The PBMC proteome of 83 subjects (N/H, *n* = 30; C/A, *n* = 25; C/S, *n* = 28) was generated as described in Materials and Methods. Proteome data sets were subjected to bipartite network analysis to identify protein spots that were univariably significant between C/A versus N/H (A), C/S versus N/H (B), and C/S versus C/A (C) subjects. The protein spots differentially abundant between C/S versus C/A subjects were also subjected to identify the protein spots that were significantly associated with disease state. Proteins/peptides distinguishing C/A versus N/H and C/S versus N/H are listed in Table S1.

We have employed a three-prong approach to identify and test the biomarkers of risk of CD progression. First, proteome data sets from the N/H, C/A, and C/S groups (19) were subjected to bipartite network analysis to identify differentially abundant proteins in CD. Of the 635 protein spots analyzed, 194 and 208 spots were univariably significant in the C/A and C/S groups, respectively (versus N/H controls, *P* < 0.001) after false-discovery rate (FDR) correction (Fig. 1A and B; see Table S1 in the supplemental material). When comparing the C/A versus C/S group, 14 proteins were differentially abundant (*P* < 0.05) and grouped in four biclusters (Fig. 1C). These protein spots were subjected to enrichment analysis by (i) splitting the patients into two random halves 1,000 times, (ii) conducting feature selection on both halves for each iteration, and (iii) identifying the top proteins that were most frequent in replicating. Cluster 4 had a significantly higher proportion of C/A subjects than the remaining subjects in the network, and they were strongly associated with fibrinogen-α, parathyroid hormone 2 receptor, myosin regulatory light chain 12B, and hnRNPA1 (*P* ≤ 0.05). Cluster 1 showed a significantly higher proportion of C/S subjects that were strongly associated with keratin 10, ATP synthase F_1_ subunit α, SH3 domain binding glutamate-rich protein 3, and vimentin (*P* ≤ 0.05). The hnRNPA1 and vimentin proteins were most stable in their association with the C/A and C/S groups, respectively.

**FIG 2:**
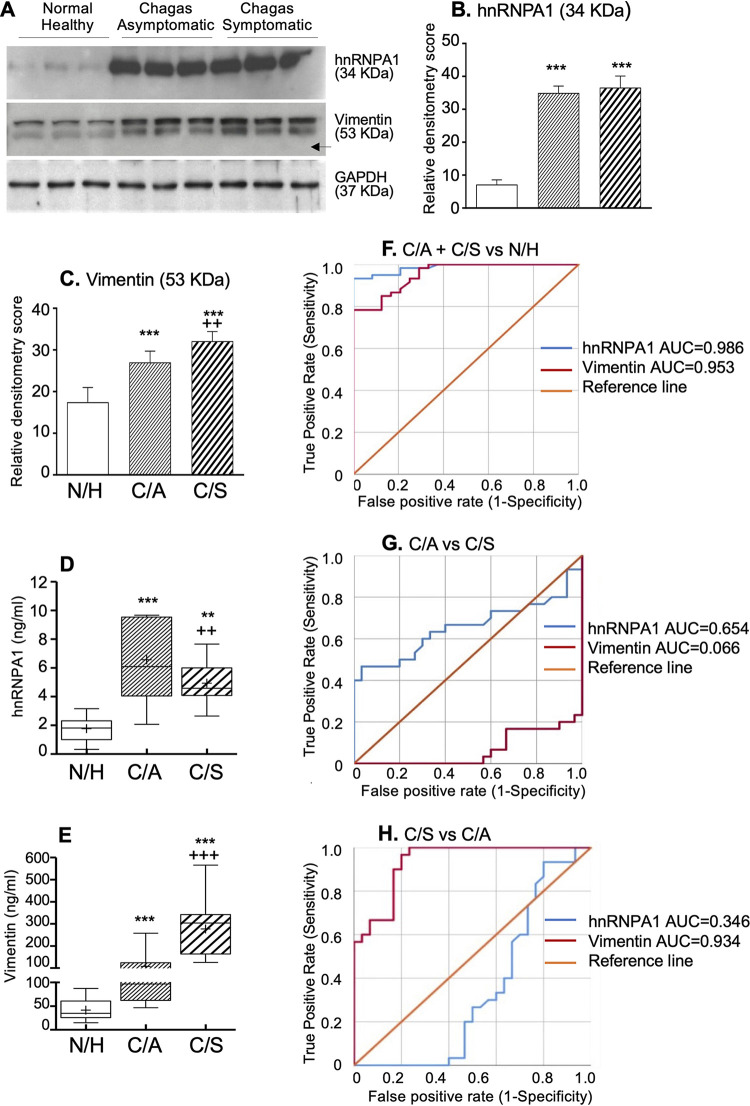
Blood levels of vimentin and hnRNPA1 in CD. A new batch of patients (*n* = 84) were recruited to perform biomarker analysis. Representative Western blot images for hnRNPA1 and vimentin (A) and their relative densitometry scores normalized to GAPDH (B and C) in PBMC homogenates are shown. Data in bar graphs are plotted as the mean value ± SD and are representative of duplicate observations per sample (*n* = 27 per group, analyzed as a pool of 3 per lane). Box plots show a quantitative estimation of serum/plasma levels of hnRNPA1 (D) and vimentin (E), determined by ELISA (N/H, *n* = 24; C/A, *n* = 30; C/S, *n* = 30). The horizontal lines of the box (bottom to top) depict the lower quartile (Q1; lowest 25%), median (Q2; middle value), and upper quartile (Q3; highest 25%). The lower and upper whiskers depict the smallest and largest nonoutlier observations, respectively. A + sign within the box plot shows the mean value. The spacing between different parts of the box indicates the degree of dispersion. Significance in bar/box graphs is presented as * for N/H versus C/A or C/S (one-way ANOVA with Tukey’s *post hoc* correction) and + for C/A versus C/S (Mann-Whitney U or Student’s two-tailed *t* test) and annotated with one symbol (* or +) for *P* ≤ 0.05, two symbols for *P* ≤ 0.01, or three symbols for *P* ≤ 0.001. (F to H) Receiver operating characteristic (ROC) curve analyses for secreted hnRNPA1 and vimentin in C/A plus C/S versus N/H (F), C/A versus C/S (G), and C/S versus C/A (H) subjects are shown.

New batches of patient samples were analyzed to verify hnRNPA1 and vimentin associations with disease severity. PBMC levels of hnRNPA1 and vimentin were increased by 3.9- to 4.2-fold and 0.6- to 0.9-fold, respectively, in C/A and C/S (versus N/H) subjects, determined by Western blotting. However, levels of PBMC hnRNPA1 were not significantly different between the C/A and C/S groups, and PBMC vimentin was increased by 20% only in the C/S (versus C/A) group (Fig. 2A to C). Secreted levels of hnRNPA1 and vimentin were increased by 1.8- to 2.7-fold and 1.6- to 5.7-fold, respectively, in C/A and C/S (versus N/H) subjects (*P* < 0.001) (Fig. 2D and E) and exhibited excellent predictive value in identifying occurrence of infection (area under the receiver operating characteristic [ROC] concentration-time curve [AUC] = 0.953 to 0.986, Cox and Snell *R*^2^ [*R*^2^_C&S_] = 0.518 to 0.598) (Fig. 2F and Table 1; see Table S2 in the supplemental material). Secreted hnRNPA1 was highest in the C/A group, and secreted vimentin was highest in the C/S group (*P* < 0.01) (Fig. 2D and E). Secreted hnRNPA1 was an independent predictor of asymptomatic CD (AUC = 0.654), and vimentin was an excellent predictor of symptomatic CD (AUC = 0.934, *R*^2^_C&S_ = 0.496) (Fig. 2G and H and Table 1; Table S2).

**TABLE 1:** Prognostic value of the biomarkers in distinguishing T. cruzi infection and CD severity^*a*^

Independent variable	N/H vs C/A + C/S (CD)				C/S vs C/A			
	*R* ^2^_NK_	*R* ^2^_C&S_	Exp(B) (95% CI)	*P* value	*R* ^2^_NK_	*R* ^2^_C&S_	Exp(B) (95% CI)	*P* value
hnRNPA1	**0.857**	**0.598**	21.982 (4.01–120.5)	0.003	0.173	0.130	0.698 (0.535–0.910)	0.005
Vimentin	**0.742**	**0.518**	1.078 (1.034–1.124)	0.001	**0.661**	**0.496**	1.025 (1.012–1.039)	0.001
8-OHdG	**0.978**	**0.6812**	7.148 (0.525–97.29)	0.001	**0.657**	**0.492**	2.152 (1.461–3.169)	0.001
PARP1	**0.992**	**0.698**	2.072 (0.034–25.76)	0.001	0.015	0.012	1.003 (0.996–1.011)	0.404
Copeptin	**0.997**	**0.718**	1.153 (0.063–24.924)	0.001	**0.918**	**0.688**	1.013 (1.004–1.023)	0.001
Endostatin	**0.665**	**0.464**	1.006 (1.003–1.009)	0.001	**0.572**	**0.432**	1.004 (1.002–1.006)	0.002
Myostatin	0.141	0.099	0.972 (0.953–0.991)	0.033	**0.954**	**0.716**	0.542 (0.321–0.916)	0.001

a Binary logistic regression analysis was performed to predict the relationship between secreted levels of independent variables and the predicted variable (i.e., disease distribution). Cox and Snell *R*^2^ (*R*^2^_C&S_) is based on log likelihood for the model compared to the log likelihood for a baseline model that has a theoretical maximum value of less than 1. Nagelkerke *R*^2^ (*R*^2^_NK_) is an adjusted version of the *R*^2^_C&S_ that adjusts the scale of the statistic to cover the full range from 0 to 1. An *R*^2^_NK_ value of >0.6 and *R*^2^_C&S_ value of >0.4 were considered significant at *P* < 0.05 and are shown in boldface. An independent variable’s association with disease state was calculated by odds ratio, denoted by exponentiation of the B coefficient [Exp(B)] with 95% CI. Exp(B) values of >1 and <1 denote positive and negative associations, respectively.

### Prognostic performance of PARP1 and 8-OHdG in CD.

**FIG 3:**
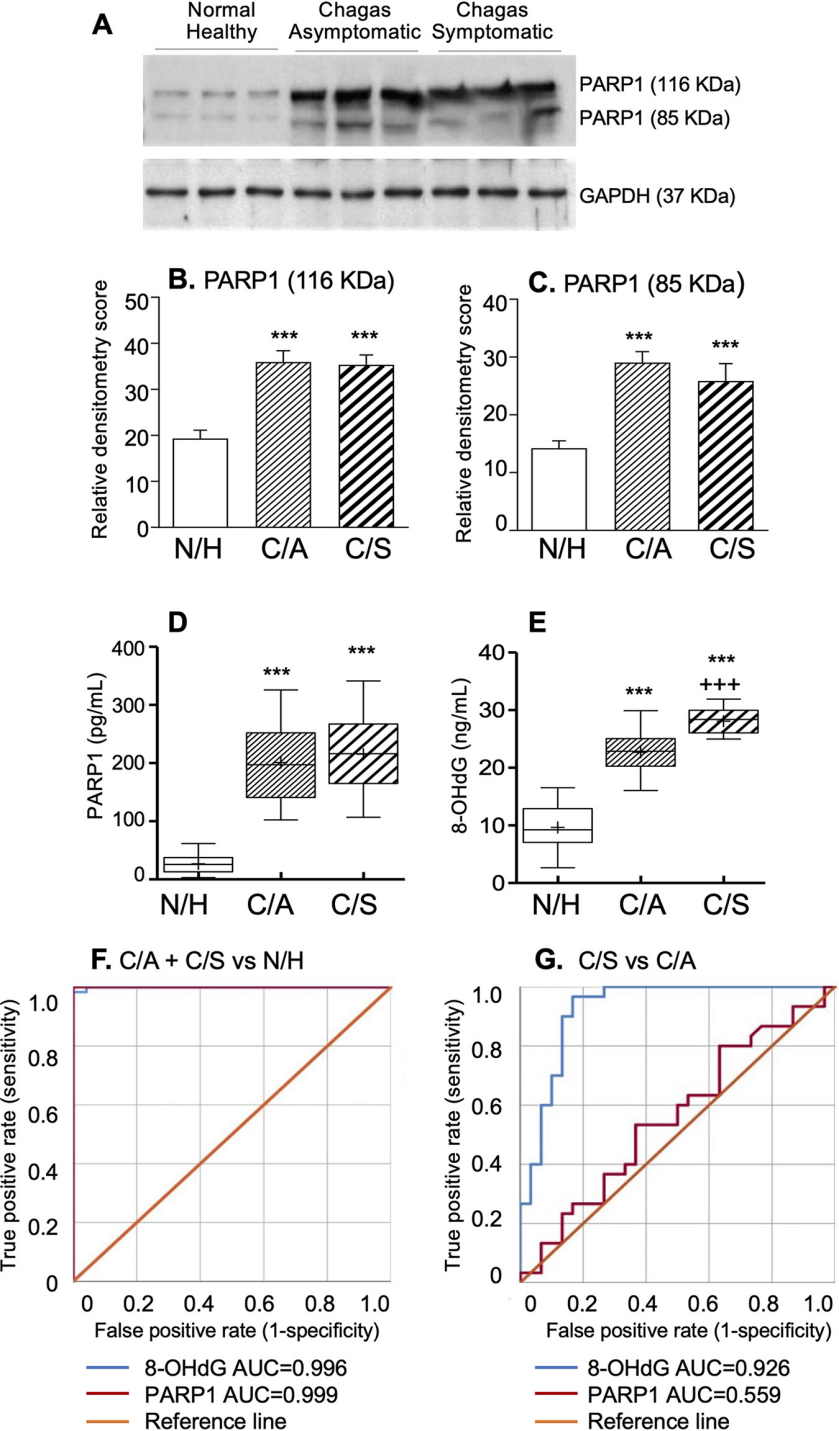
Blood levels of PARP1 and 8-OHdG in CD. Representative Western blot images for full-length and cleaved/active forms of PARP1 (A) and their relative densitometry score normalized to GAPDH (B and C) in PBMC homogenates are plotted as the mean value ± SD (*n* = 27 per group, analyzed as a pool of 3 per lane). Box plots show serum/plasma levels of PARP1 (D) and 8-OHdG (E), determined by ELISA (N/H, *n* = 24; C/A, *n* = 30; C/S, *n* = 30). ROC analyses for secreted PARP1 and 8-OHdG in CD versus N/H (F) and C/S versus C/A (G) subjects are shown. Details of box plot depiction and significance are presented in Fig. 2.

PBMC levels of the 116-kDa PARP1 (full-length) and 89-kDa PARP1 (active form) were increased by 80 to 85% and 85 to 110%, respectively, in C/A and C/S subjects (versus N/H; *P* < 0.001), respectively. No significant differences were noted in PBMC PARP1 levels between the C/A and C/S groups (Fig. 3A to C). Secreted PARP1 and 8-OHdG levels were increased by 6.5- to 7.0-fold and 1.4- to 1.9-fold, respectively, in C/A and C/S subjects (versus N/H; *P* < 0.001) (Fig. 3D and E) and exhibited strong association as independent predictors of occurrence of infection (AUC ≥ 0.99; PARP1 *R*^2^_C&S_ = 0.698; 8-OHdG *R*^2^_C&S_ = 0.682) (Fig. 3F and Table 1; Table S2). When comparing the C/A and C/S groups, secreted PARP1 levels were not discriminatory, while secreted 8-OHdG levels were higher in C/S (versus C/A) subjects (*P* < 0.001) (Fig. 3E) and exhibited excellent capability in distinguishing symptomatic from asymptomatic disease (AUC = 0.926; *R*^2^_C&S_ = 0.492) (Fig. 3G and Table 1; Table S2).

### Predictive values of copeptin, endostatins, and myostatin in CD.

**FIG 4:**
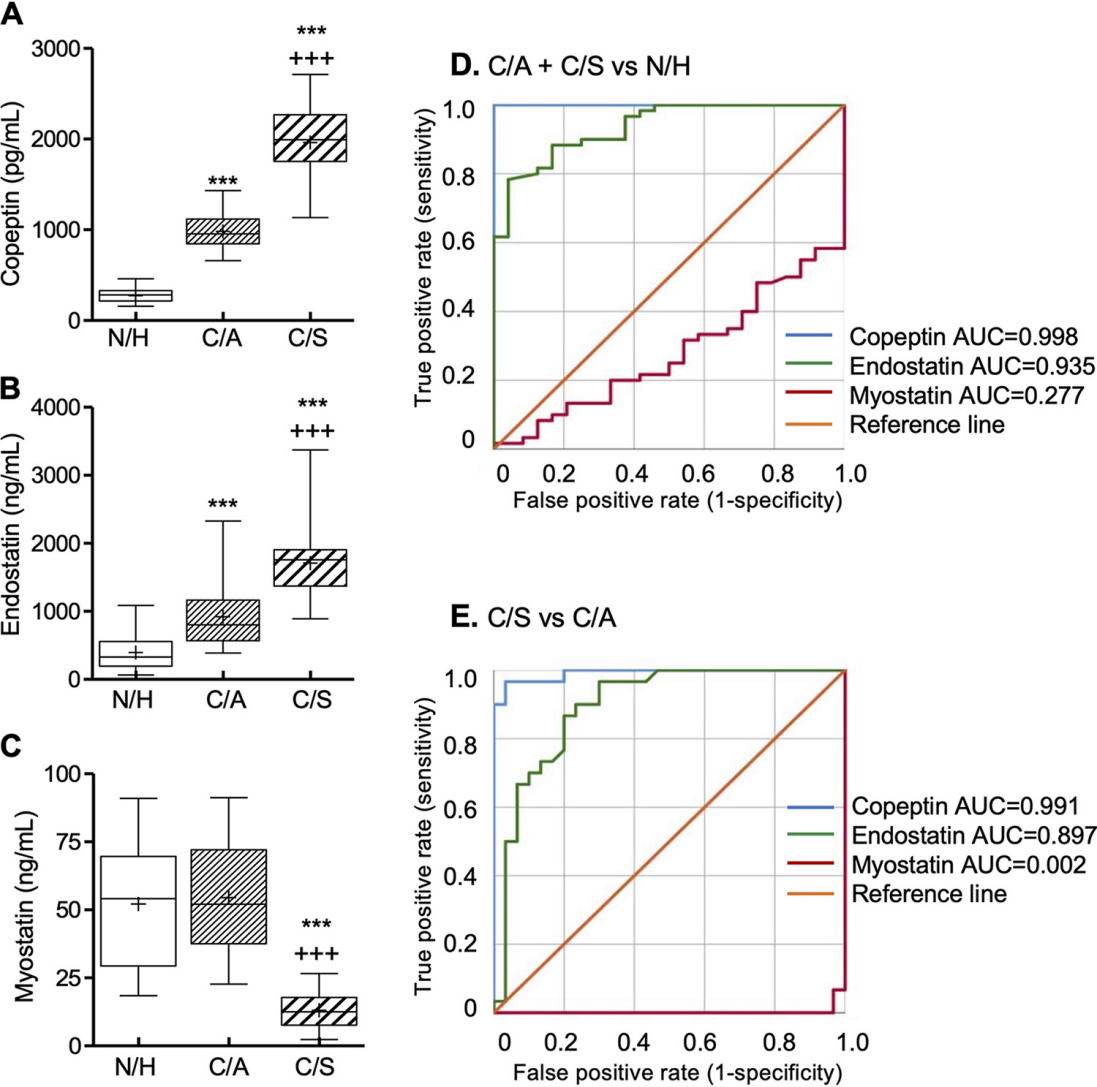
Blood levels of cardiac dysfunction markers in CD. Box plots show serum/plasma levels of copeptin (A), endostatin (B), and myostatin (C), determined by ELISA (N/H, *n* = 24; C/A, *n* = 30; C/S, *n* = 30). ROC analyses for secreted copeptin, endostatin, and myostatin in C/A plus C/S versus N/H (D) and C/S versus C/A (E) subjects are shown. Details of box plot depiction and significance are presented in Fig. 2.

Serum/plasma levels of copeptin and endostatin were increased by 2.6- to 6.2-fold and 1.4- to 3.4-fold, respectively, in C/A and C/S (versus N/H) individuals (*P* < 0.001), the highest levels being detected in C/S subjects (*P* < 0.001) (Fig. 4A and B). Both copeptin (AUC = 0.998; *R*^2^_C&S_ = 0.718) and endostatin (AUC = 0.935; *R*^2^_C&S_ = 0.464) exhibited high performance in predicting occurrence of infection (Fig. 4D and Table 1). More importantly, excellent performance of copeptin (AUC = 0.991; *R*^2^_C&S_ = 0.688) and good performance of endostatin (AUC = 0.897; *R*^2^_C&S_ = 0.432) in distinguishing symptomatic from asymptomatic CD were noted (Fig. 4E and Table 1; Table S2). Secreted myostatin levels were not changed between C/A and N/H groups, while a 75 to 76% decline in myostatin release occurred in symptomatic CD patients (* or +, *P* < 0.001) (Fig. 4C). Consequently, myostatin was not good predictor of infection (Fig. 4D); however, its decline was a strong independent predictor of C/A (versus C/S) CD (AUC = 0.998; *R*^2^_C&S_ = 0.716) (Table 1; Table S2).

### Serum and plasma are equally useful in biomarker analysis.

We separately analyzed the biomarkers’ levels in serum and plasma to determine if either of the blood components is better quantitatively or qualitatively. We didn’t observe significant differences in the biomarkers’ levels when comparing serum and plasma from the same patient in any of the studied groups (see Fig. S1 in the supplemental material). Thus, both serum and plasma can be used for biomarker analysis in distinguishing CD severity.

### Cumulative distribution pattern of biomarkers with CD progression.

Cumulative patients’ data for the independent variables are shown in Fig. S2 in the supplemental material. A two-dimensional radar chart allows rapid comparison of the biomarkers’ distribution pattern throughout the spectrum of asymptomatic to symptomatic CD in comparison to healthy controls. This analysis shows vimentin, copeptin, endostatin, 8-OHdG, and PARP1 were maximally increased in CD (versus N/H), vimentin and copeptin are maximally different between the C/A and C/S groups, and the decline in myostatin is associated with C/S (versus C/A or N/H) prediction.

### Biomarkers’ performance in predicting risk of cardiac dysfunction.

Finally, we used an ejection fraction (EF) of <55% and diastolic diameter of >57 mm as dependable markers of clinically symptomatic disease and determined if the studied biomarkers predict the risk of left ventricular (LV) dysfunction in CD. We noted a strong association of increase in secreted 8-OHdG (Wald χ^2^ = 15.065), vimentin (Wald χ^2^ = 14.587), and endostatin (Wald χ^2^ = 17.902) levels with decline in EF and increase in diastolic diameter in CD patients (Table 2). Secreted copeptin levels (Wald χ^2^ = 7.618) were also positively associated with symptomatic disease parameters. Furthermore, increase in secreted hnRNPA1 (Wald χ^2^ = 7.084) was positively associated with >55% EF and <57-mm diastolic diameter indicative of C/A CD. Other biomarkers were not conclusive in identifying the risk of disease level in CD patients. These results suggest that higher levels of secreted endostatin, 8-OHdG, and vimentin indicate increased risk of symptomatic CD development, while higher levels of circulating hnRNPA1 indicate lower risk of symptomatic disease in T. cruzi-infected humans.

**TABLE 2:** Predictive value of biomarkers in identifying risk of cardiomyopathy development in CD subjects^*a*^

Independent variable	Ejection fraction			Diastolic diameter		
	Wald χ^2^	Exp(B) (95% CI)	*P* value	Wald χ^2^	Exp(B) 95% CI	*P* value
hnRNPA1	7.084	1.433 (1.099–1.868)	0.008	7.084	0.698 (0.535–0.910)	0.008
Vimentin	14.587	0.975 (0.963–0.988)	0.001	14.58	1.025 (1.012–1.069)	0.001
PARP1	0.689	0.997 (0.979–1.004)	0.689	0.689	1.003 (0.996–1.011)	0.406
8-OHdG	15.065	0.465 (0.316–0.684)	0.001	15.065	2.152 (1.461–3.169)	0.001
Copeptin	7.618	0.987 (0.978–0.996)	0.006	7.618	1.018 (1.002–1.032)	0.006
Endostatin	17.902	0.996 (0.990–0.998)	0.001	17.902	1.008 (1.003–1.012)	0.001
Myostatin	5.228	1.845 (1.091–3.118)	0.022	5.228	0.540 (0.321–0.916)	0.022

a Binary logistic regression analysis with the Wald test was performed by assuming ejection fraction and diastolic diameter as dichotomous dependent variables to assess whether independent variables can predict the risk of cardiomyopathy development in CD. Wald χ^2^ test values of >5 were considered significant at *P* < 0.05. An independent variable’s association with disease state was calculated by odds ratio denoted by exponentiation of the B coefficient [Exp(B)] with 95% CI. Exp(B) values of >1 and <1 denote positive and negative associations, respectively. An ejection fraction of <55% and a diastolic diameter of ≥57 mm were considered to be indicative of clinically symptomatic, while an ejection fraction of >55% and diastolic diameter of <57 mm were considered be indicative of a clinically asymptomatic state in CD patients.

## DISCUSSION

With reference to T. cruzi infection and CD, we need validated diagnostic methods to identify (i) parasite exposure, (ii) treatment efficacy, and (iii) risk of clinical disease development. Tests for diagnosis of T. cruzi infection that are used in clinical and research settings include serology, PCR, and xenodiagnosis. These tests offer significant accuracy in determining the acute to chronic exposure to parasite (28). The “gold standard” for monitoring the efficacy of antiparasite treatments and cure is seroconversion, which can take decades to occur (29, 30). Others have investigated reactivity to parasite (e.g., mucins) or host (e.g., apolipoprotein A1 or fibronectin) antigens, and circulating parasite DNA levels to estimate the treatment efficacy of benznidazole and nifurtimox in CD patients. Because low levels of blood parasites are intermittently detected in the chronic phase, multiple assessments of parasite antigens and/or DNA are recommended to ensure treatment efficacy. Overall, there is a lack of validated tools for evaluating the therapeutic responses of patients with chronic infection to established or newly developed drugs and vaccines. Furthermore, our toolbox to evaluate the efficacy of a treatment in a short period of time and precisely inform the patients about their therapeutic progress is almost empty.

Cytokines have been proposed as biomarkers of disease state in several infectious diseases. In CD, IFN-γ, IL-2, IL-6, IL-10, IL-12, and IL-17 are proposed as surrogate markers of cardiac disease (versus no disease) (9–14). However, these immune molecules play an important role in balancing proinflammatory and anti-inflammatory responses so the host can clear T. cruzi while not sustaining self-tissue injury. Thus, cytokines may not provide an independent biomarker of disease state (versus host response to infection). Cardiological markers (ACE2, BNP, and troponin) or liver enzymes may also offer indications of CD progression. Liver enzymes have not been assessed in seropositive CD individuals, and ACE2 and BNP levels were increased in CD patients with heart failure (HF) (15, 16), as is also observed in HF of other etiologies. However, the prognostic value of the above-discussed biomarkers in predicting progression of symptomatic heart disease in CD remains undefined. Finding simple quantifiable biomarkers that are consistently reliable in conventional screening and can be routinely used in prognosis and diagnosis of CD patients at risk of developing cardiomyopathy and HF remains challenging so far.

The present study was intended to evaluate the prognostic performance of new biomarkers in distinguishing the clinical state of CD and identifying the risk of CD progression. To the best of our knowledge, with exception of vimentin (17, 18), none of the studied biomarkers has previously been evaluated in CD.

hnRNPA1 is an RNA-binding protein involved in regulating collagen synthesis. Increased hnRNPA1 expression in heart muscle of patients experiencing aortic stenosis, ischemic cardiomyopathy, or dilated cardiomyopathy is noted (31), although its release in circulation was not examined. Vimentin, a type III intermediate filament protein, is found in many cells. Increases in circulatory antivimentin antibodies and vimentin peptides have been proposed as biomarkers of T. cruzi infection and muscle damage, respectively, in CD (17, 18, 32). In this study, we noted increased vimentin and hnRNPA1 secretion in seropositive CD subjects. Vimentin exhibited excellent prognostic sensitivity in identifying symptomatic CD, while hnRNPA1 was more promising prognostic marker of asymptomatic CD. These two biomarkers were identified by bipartite network analysis of the proteome data sets. The value of this approach is to show not only which single biomarker occurs in different clusters enriched by cases or controls, but also how they frequently co-occur with other biomarkers. We noted that an increase in hnRNPA1 in C/A subjects frequently co-occurred with parathyroid hormone 2 receptor and myosin regulatory light chain, and an increase in vimentin in C/S subjects co-occurred with keratin 10 and the ATP synthase F_1_ subunit. These co-occurrences likely provide insights into potential underlying pathways in each cluster (i.e., disease severity group), to be investigated in future studies.

PARP1 responds to oxidative DNA lesions (e.g., 8-OHdG). We have documented a regulatory role of PARP1 in transcriptional activation of profibrotic and proinflammatory gene expression in T. cruzi infection (22), and PARP1 depletion improved the LV function in chronically infected mice (23). An increase in PARP1 activity in the failing heart was linked to coronary artery disease in diabetes patients (33, 34). Likewise, secreted 8-OHdG levels were increased in cardiomyopathy, HF, and cardiac sarcoidosis patients (35–37). We detected significantly higher levels of PARP1 and 8-OHdG in serum/plasma and the PARP1 active form in PBMCs of CD subjects. While both markers showed promising prognostic performance in detection of infection, only 8-OHdG had the potential to serve as a biomarker in differentiating symptomatic from asymptomatic CD.

Copeptin and endostatin have emerged as new biomarkers for diagnosing cardiac involvement, myocardial infarction, and HF events. Plasma copeptin levels exhibited higher clinical applicability in prediction of HF in patients with reduced left ventricular ejection fraction (38) or hyponatremia symptoms (39) and in prediction of all-cause death and readmission in HF patients (40). Serum endostatin levels offered clinically relevant association with LV diastolic dysfunction and inhibition of adaptive angiogenesis in HF patients (41). Serum endostatin levels also correlated with cardiogenic events in patients presenting unstable angina pectoris and stable coronary artery disease (42). Secreted copeptin and endostatin exhibited excellent and good prognostic sensitivity in distinguishing symptomatic and asymptomatic CD. Myostatin is a negative regulator of muscle growth that causes muscle atrophy (43). Increased myocardial expression of myostatin was noted in congenital heart disease, and an increase in the ratio of myostatin to insulin-like growth factor 1 correlated with worsening of LV function (44). Our findings of significantly low myostatin levels in serum of C/S CD patients are in alignment with those of Furikata et al. (45), who noted decreased serum levels of myostatin were associated with lower-extremity muscle wasting in HF patients.

Impaired systolic function and increased ventricular dimensions have significant value in predicting cardiac morbidity and mortality in CD patients (46), and these parameters were employed to distinguish the C/A versus C/S stage of CD in patients enrolled in this study. Others have documented that changes in right ventricular function and left atrial volume, along with decline in left ventricular ejection fraction, predict adverse outcomes in CD (47). The survival prognosis of CD patients with chronic systolic HF was poorer than that seen in ischemic heart disease patients (48). Our data show a significant relationship between secreted levels of vimentin, 8-OHdG, and endostatin with decreased EF and increased diastolic diameter in CD patients and offer new blood biomarkers to predict the risk of cardiomyopathy, HF, and adverse outcomes in CD patients. Conversely, higher levels of circulating hnRNPA1 indicated reduced risk of symptomatic CD development in infected individuals.

Summarizing, our results suggest that plasma/serum levels of four biomarkers (vimentin, 8-OHdG, copeptin, and endostatin) will be useful in distinguishing symptomatic from asymptomatic CD and in monitoring the risk of increase in CD severity in infected individuals. A decline in these four biomarkers along with an increase in hnRNPA1 levels can also be used for monitoring the efficacy of therapies in controlling symptomatic CD. Of note, these biomarkers can easily be screened using the readily available plasma/serum samples in a clinical setting by an enzyme-linked immunosorbent assay (ELISA). Serum/plasma samples need to be stored at 4°C only, and ELISAs are inexpensive, fast, and require low-tech resources at the facility, equipment, and personnel levels. Furthermore, ELISAs are amenable to large-scale sample analysis to obtain maximum efficiency at the personnel and cost levels. We hope clinician can use these markers as a routine parameter to assess the clinical severity of CD.

One limitation of this study is that selected biomarkers were analyzed in Chagas patients from northern Argentina, where T. cruzi isolates of lineage II (TcII) primarily circulate in the vector and the mammalian hosts. Future studies enrolling larger cohorts of patients from different areas of endemicity along with meta-analysis will be needed to establish the normal reference range and threshold levels of these biomarkers for distinguishing the Chagas disease state and severity in patients exposed to various T. cruzi lineages. Likewise, longitudinal screening of the Chagas patients with documentation of clinical history will evaluate the extent by which the comorbid diseases (i.e., diabetes, coronary artery disease, and recent microbial infection) may further elevate the secreted levels of selected biomarkers and increase the risk of heart failure in Chagas patients.

## MATERIALS AND METHODS

### Ethics statement.

Human subject protocol (13-0367) was approved by institutional review board (UTMB Galveston) and ethics committee (UNSa, Salta, Argentina). Subjects with preexisting clinical conditions (e.g., diabetes, coronary artery disease, or recent viral or bacterial infections) were excluded. Written informed consent was obtained, and samples were decoded and deidentified for research use.

### Human samples.

In the discovery phase, proteome studies were performed using PBMC samples from 83 subjects (N/H, *n* = 30; C/A, *n* = 25; C/S, *n* = 28) as described previously (19).

The demographics of the newly recruited 84 subjects (N/H, *n* = 24; C/A, *n* = 30; C/S, *n* = 30) in this study are presented in Table S3 in the supplemental material. Blood samples were obtained from consenting adults visiting Hospital San Bernardo, Salta, Argentina, during January through December in 2016 to 2018. Subjects positive by the Chagatest-ELISA and Chagatest-HAI kits (Wiener) were considered seropositive. Routine physical exams, electrocardiography, and transthoracic echocardiography were performed to assess frequency or severity of exertional dyspnea and left ventricular (LV) function. Seronegative individuals with no cardiac involvement were categorized as noninfected, healthy (N/H). Seropositive subjects with no to minor echocardiography abnormalities, no LV dilatations, and normal ejection fraction (EF, 55 to 70%) were grouped as clinically asymptomatic (C/A). Seropositive individuals with systolic dysfunction (EF, <55%), LV dilatation (diastolic diameter, ≥57 mm), and/or signs of congestive heart failure were grouped as clinically symptomatic (C/S).

Whole-blood samples (6 ml) were collected in BD-Vacutainer tubes with K_2_EDTA anticoagulant or without anticoagulant to obtain plasma and serum, respectively. One hour after collection, samples were centrifuged at 4,000 rpm for 30 min. Plasma and serum samples were aspirated and stored at −80°C. Another batch of blood samples were processed using Histopaque-1077 separation medium (Sigma-Aldrich) and density gradient centrifugation at 400 × *g* for 10 min to purify PBMCs, which were then aspirated into a 15-ml tube, washed with phosphate-buffered saline (PBS), and stored at −80°C. PBMCs were resuspended in PBS for use.

### Proteome analysis and bipartite network analysis.

PBMC samples from 83 enrolled humans (N/H, *n* = 30; C/A, *n* = 25; C/S, *n* = 28) were subjected to proteome analysis as described previously (19). Briefly, samples were resolved by two-dimensional electrophoresis, and gel images were analyzed by Same Spots software. Protein spot volumes were normalized, log transformed, and analyzed in a pairwise manner by *t* test with Welch’s correction, which yielded 635 differentially abundant protein spots (≥|1.5|-fold change, *P* < 0.05 with Benjamini-Hochberg correction) in CD (versus N/H) subjects. Protein spots were analyzed by matrix-assisted laser desorption ionization–time of flight mass spectrometry (MALDI-TOF MS), and data were interrogated against the UniProt human proteome database for protein identification. The proteome data sets were previously described (19). Log-transformed proteome data sets were subjected to bipartite network analysis and visualization (49) using the following steps: (i) feature selection to identify which protein spots were univariably significant (after FDR correction) between different comparison groups; (ii) bipartite modularity maximization (50) to identify biclusters of patients and protein spots, with testing of significance through comparison to biclustering generated from 1,000 permutations of the data; and (iii) enrichment analysis using χ^2^ to determine if there was a significantly different proportion of phenotypes in each bicluster compared to the rest of the data.

### Western blotting.

PBMCs purified from 6-ml blood samples were washed with ice-cold phosphate-buffered saline (PBS) and suspended at a cell/buffer ratio of 1:10 (wt/vol) in 1× radioimmunoprecipitation assay (RIPA) buffer (20 mM Tris-HCl [pH 7.5], 150 mM NaCl, 1 mM Na_2_EDTA, 1 mM EGTA, 1% NP-40, 1% sodium deoxycholate, 2.5 mM sodium pyrophosphate, 1 mM β-glycerophosphate, 1 mM Na_3_VO_4_, 1 μg/ml leupeptin, and 1 mM phenylmethylsulfonyl fluoride [PMSF]). PBMCs were then lysed by 3 freeze-thaw cycles (30 min each) followed by sonication 3 times (20 s each) by using an XL Ultrasonic Processor sonicator (Misonix, NY). Cell homogenates were centrifuged at 14,000 × *g* at 4°C for 10 min, and protein concentrations in cleared supernatants were determined by using the Bio-Rad Bradford protein assay.

PBMC cleared lysates (*n* = 27 per group, analyzed as a pool of 3 per lane) were resolved on 10% polyacrylamide gel using a Mini-PROTEAN electrophoresis system (Bio-Rad), and proteins (25 μg) were transferred to polyvinylidene difluoride (PVDF) membrane using a Criterion Trans-Blot device (Bio-Rad). Membranes were blocked for 2 h with 20 mM Tris-HCl (pH 7.4)–136 mM NaCl containing 0.5% bovine serum albumin (BSA) and 0.1% Tween 20 (TBST) and incubated overnight with anti-hnRNPA1 (ab5832), antivimentin (ab8978), or anti-PARP1 (ab227244) antibodies (1:1,000 dilution; Abcam) and for 1 h with horseradish peroxidase (HRP)-conjugated secondary antibody (1:5,000 dilution; Southern Biotech). TBST was used for all dilutions. Color was developed using Pierce ECL enhanced chemiluminescence substrate, and images were acquired using an Image Quant LAS4000 (GE Healthcare). Protein bands of interest were analyzed and normalized against glyceraldehyde-3-phosphate dehydrogenase (GAPDH) levels using Image J software (National Institutes of Health).

### Enzyme-linked immunosorbent assay.

The enzyme-linked immunosorbent assay (ELISA) was performed using plasma and serum samples (N/H, *n* = 24; C/A, *n* = 30; C/S, *n* = 30). ELISA kits from Abcam (8-OHdG, ab201734; endostatin, ab100508; myostatin, ab267656) or MyBiosource (hnRNPA1, MBS732451; vimentin, MBS355298; PARP1, MBS703546; copeptin, MBS703328) were used following the manufacturers’ instructions. Standard curves were prepared by using recombinant proteins (sensitivities of 1 ng/ml vimentin, 0.1 ng/ml hnRNPA1, 3.9 pg/ml PARP1, 1 pg/ml 8-OHdG, 19.5 pg/ml copeptin, 0.83 ng/ml myostatin, and 10 pg/ml endostatin). The intra- and interassay variability values were 2.1 to 5.3 and 2.8 to 4.5, respectively, for the various assays.

### Statistical analysis.

Data were analyzed using GraphPad Prism (v.9) and IBM SPSS (v.25) software. Preliminary mean ± standard deviation (SD) values for each independent group were used to evaluate the statistical power of sample size. By considering the CD incidence rate of 4.1% in the studied population, we calculated that 54 seropositive subjects would offer a 95% confidence interval (CI) to achieve a statistical power of 80% with type 1 error at the 0.05 level. The conformity of continuous variables to the normal distribution was evaluated by Kolmogorov-Smirnov test, and data were analyzed by Mann-Whitney U test or Student’s two-tailed unpaired *t* test to compare the means of two groups and by nonparametric Kruskal-Wallis test or one-way analysis of variance (ANOVA) when comparing three groups. Data were plotted in bar graph, box plot, or radar formats.

The predictive prognostic value of biomarkers was evaluated by the area under the receiver operating characteristic (ROC) curve (AUC). By assuming a binary model of disease distribution—i.e., N/H (0) versus CD (1) and C/S (1) versus C/A (0)—logistic regression analysis was performed. Cox & Snell and Nagelkerke coefficients of determination (*R*^2^_C&S_ and *R*^2^_NK_, respectively) were calculated to measure how close data are fitted to the regression line and depict the relationship between the independent and predicted variables. An *R*^2^_NK_ value of >0.6 and *R*^2^_C&S_ value of >0.4 were considered significant at *P* < 0.05. Binary logistic regressions with the Wald test were performed to test if independent variables are in significant association with dichotomous dependent variables (EF and diastolic diameter) and predict the risk of cardiomyopathy development in CD patients. A Wald χ^2^ value of >5 was considered significant at *P* < 0.05. The contribution of each independent variable in the model and their significant association with disease state were calculated by odds ratio denoted by exponentiation of B coefficients [Exp(B)] with 95% CI. Exp(B) values of >1 and <1 signify positive and negative associations, respectively.

### Data availability.

All relevant data are included in the article.
