# Supplementary material for: Prognostic Performance of Peripheral Blood Biomarkers in Identifying Seropositive Individuals at Risk of Developing Clinically Symptomatic Chagas Cardiomyopathy
Source: Microbiol Spectr. 2021 Aug 25;9(1):10.1128/spectrum.00364-21. doi: 10.1128/spectrum.00364-21 (PMC8552597; doi:10.1128/spectrum.00364-21)
Supplement: SUPPLEMENTAL FILE 1 — Supplemental material. Download SPECTRUM00364-21_Supp_1_seq9.pdf, PDF file, 1.3 MB [file spectrum00364-21_supp_1_seq9.pdf]

Supplemental Material for Publication

Table S1: Bipartite network analysis and identification of peptides that were univariably significant in Chagas vs. healthy subjects.

Clinically Asymptomatic (C/A)		Non-infected Healthy (N/H)	
**V617**	Actin, alpha 1, skeletal muscle, GN=ACTA1--890--E3	**V165**	60 kDa heat shock protein, mitochondrial, GN=HSPD1--257--I3
**V221**	Actin, cytoplasmic 1, GN=ACTB--321--D5	**V54**	Actin, alpha 1, skeletal muscle, GN=ACTA1--121--I21
**V232**	Actin, cytoplasmic 1, GN=ACTB--335--D6	**V39**	Actin, cytoplasmic 1, GN=ACTB--101--K16
**V234**	Actin, cytoplasmic 1, GN=ACTB--337--D7	**V80**	Actin, cytoplasmic 1, GN=ACTB--154--H24
**V345**	Actin, cytoplasmic 1, GN=ACTB--479--D11	**V99**	Actin, cytoplasmic 1, GN=ACTB--180--J11
**V461**	Actin, cytoplasmic 1, GN=ACTB--621--A11	**V103**	Actin, cytoplasmic 1, GN=ACTB--184--J12
**V465**	Actin, cytoplasmic 1, GN=ACTB--629--A13	**V104**	Actin, cytoplasmic 1, GN=ACTB--185--K19
**V473**	Actin, cytoplasmic 1, GN=ACTB--640--13	**V144**	Actin, cytoplasmic 1, GN=ACTB--232--G15
**V475**	Actin, cytoplasmic 1, GN=ACTB--642--E15	**V149**	Actin, cytoplasmic 1, GN=ACTB--238--J16
**V485**	Actin, cytoplasmic 1, GN=ACTB--657--A14	**V155**	Actin, cytoplasmic 1, GN=ACTB--244--G18
**V489**	Actin, cytoplasmic 1, GN=ACTB--662--A16	**V162**	Actin, cytoplasmic 1, GN=ACTB--254--J17
**V492**	Actin, cytoplasmic 1, GN=ACTB--665--A17	**V171**	Actin, cytoplasmic 1, GN=ACTB--264--J18
**V500**	Actin, cytoplasmic 1, GN=ACTB--675--A18	**V175**	Actin, cytoplasmic 1, GN=ACTB--268--J19
**V501**	Actin, cytoplasmic 1, GN=ACTB--676--A19	**V177**	Actin, cytoplasmic 1, GN=ACTB--270--J20
**V502**	Actin, cytoplasmic 1, GN=ACTB--677--A20	**V182**	Actin, cytoplasmic 1, GN=ACTB--275--G22
**V545**	Actin, cytoplasmic 1, GN=ACTB--763--A24	**V205**	Actin, cytoplasmic 1, GN=ACTB--303--B10
**V547**	Actin, cytoplasmic 1, GN=ACTB--769--B1	**V12**	Actin, cytoplasmic 1, GN=ACTB--69--H16
**V623**	Actin, cytoplasmic 1, GN=ACTB--896--B5	**V539**	Actin, cytoplasmic 1, GN=ACTB--756--K8
**V624**	Actin, cytoplasmic 1, GN=ACTB--897--B6	**V550**	Actin, cytoplasmic 1, GN=ACTB--779--D24
**V454**	Actin, cytoplasmic 2, GN=ACTG1--610--D16	**V551**	Actin, cytoplasmic 1, GN=ACTB--781--E1
**V457**	Actin, cytoplasmic 2, GN=ACTG1--613--D17	**V52**	Actin, cytoplasmic 2, GN=ACTG1--118--K17
**V462**	Actin, cytoplasmic 2, GN=ACTG1--623--A12	**V163**	Actin, cytoplasmic 2, GN=ACTG1--255--I2
**V487**	Actin, cytoplasmic 2, GN=ACTG1--659--A15	**V176**	Actin, cytoplasmic 2, GN=ACTG1--269--J2
**V542**	Actin, cytoplasmic 2, GN=ACTG1--759--D22	**V145**	Alpha-enolase, GN=ENO1--233--F10
**V544**	Actin, cytoplasmic 2, GN=ACTG1--762--L14	**V146**	Alpha-enolase, GN=ENO1--234--J15
**V618**	Actin, cytoplasmic 2, GN=ACTG1--891--E4	**V151**	Alpha-enolase, GN=ENO1--240--G17
**V435**	Alpha-actinin-1, GN=ACTN1--588--A8	**V158**	Alpha-enolase, GN=ENO1--247--G19
**V372**	Apolipoprotein A-I, GN=APOA1--515--L4	**V164**	Alpha-enolase, GN=ENO1--256--G20
**V532**	ATP synthase subunit alpha, mitochondrial, GN=ATP5A1--737--	**V262**	Annexin A1, GN=ANXA1--373--H1
**V481**	Bestrophin-3, GN=BEST3--648--D1	**V134**	ATP synthase subunit beta, GN=ATP5B--219--G14
**V498**	Bestrophin-3, GN=BEST3--671--B23	**V455**	Cofilin 1 (Non-muscle), GN=CFL1--611--K5
**V510**	Centromere protein H, GN=CENPH--686--D2	**V125**	Cyclin-dependent kinase 4, GN=CDK4--207--F7
**V59**	Fibrinogen alpha chain isoform 2, GN=FGA--127--G7	**V230**	Cyclin-dependent kinase 4, GN=CDK4--333--I12
**V616**	Fibrinogen alpha chain isoform 2, GN=FGA--889--H12	**V74**	Early endosome antigen 1, GN=EEA1--147--F2
**V470**	Heterogeneous nuclear ribonucleoprotein K, GN=HNRNPK--637-	**V167**	Elongation factor Tu, mitochondrial, GN=TUFM--260--I4
**V508**	Histone H4, GN=HIST1H4A--683--A22	**V169**	Elongation factor Tu, mitochondrial, GN=TUFM--262--I5
**V513**	Histone H4, GN=HIST1H4A--696--A23	**V147**	Enolase, GN=ENO3--235--F11
**V528**	Histone H4, GN=HIST1H4A--725--E18	**V126**	Fibrinogen alpha chain isoform 2, GN=FGA--208--I24
**V548**	Histone H4, GN=HIST1H4A--772--B2	**V178**	Fibrinogen alpha chain isoform 2, GN=FGA--271--G21
**V597**	Keratin, type I cytoskeletal 9, GN=KRT9--863--E2	**V265**	Fibrinogen alpha chain isoform 2, GN=FGA--377--B15
**V559**	Keratin, type II cuticular Hb3, GN=KRT83--797--E20	**V110**	Fibrinogen beta chain, GN=FGB--191--F3
**V429**	Keratin, type II cytoskeletal 1, GN=KRT1--582--A7	**V114**	Fibrinogen beta chain, GN=FGB--195--G11
**V533**	Keratin, type II cytoskeletal 1, GN=KRT1--738--D21	**V115**	Fibrinogen beta chain, GN=FGB--196--F4
**V543**	Keratin, type II cytoskeletal 1, GN=KRT1--761--D23	**V116**	Fibrinogen beta chain, GN=FGB--197--G12
**V558**	Keratin, type II cytoskeletal 1, GN=KRT1--796--E19	**V119**	Fibrinogen beta chain, GN=FGB--200--I22
**V579**	Lactotransferrin, isoform delta GN=LTF--825--C8	**V120**	Fibrinogen beta chain, GN=FGB--201--F6
**V599**	LVV-hemorphin-7, GN=HBB--867--C11	**V121**	Fibrinogen beta chain, GN=FGB--202--I23
**V608**	LVV-hemorphin-7, GN=HBB--879--E23	**V123**	Fibrinogen beta chain, GN=FGB--205--C16
**V406**	Mitochondrial carrier homolog 1 isoform 3, GN=MTCH1--552--	**V130**	Fibrinogen beta chain, GN=FGB--213--F8
**V460**	Mitochondrial carrier homolog 1 isoform 3, GN=MTCH1--620--	**V131**	Fibrinogen beta chain, GN=FGB--214--C17
**V493**	Mitochondrial carrier homolog 1 isoform 3, GN=MTCH1--666--	**V38**	Filamin-A, GN=FLNA--100--H20
**V464**	Myosin light polypeptide 6, GN=MYL6--628--E14	**V40**	Filamin-A, GN=FLNA--102--G5
**V456**	Myosin regulatory light polypeptide 9, GN=MYL9--612--K21	**V41**	Filamin-A, GN=FLNA--103--H21
**V523**	Myotrophin, GN=MTPN--712--I17	**V30**	Filamin-A, GN=FLNA--91--H17
**V526**	Protein S100-A6, GN=S100A6--721--C5	**V31**	Filamin-A, GN=FLNA--92--F24
**V598**	Protein S100-A6, GN=S100A6--866--C10	**V32**	Filamin-A, GN=FLNA--93--H18
**V569**	Protein S100-A8, GN=S100A8--809--E22	**V33**	Filamin-A, GN=FLNA--94--G1
**V525**	Ras-related protein Ral-B, GN=RALB--718--C4	**V34**	Filamin-A, GN=FLNA--95--G2
**V557**	Ras-related protein Ral-B, GN=RALB--787--K9	**V35**	Filamin-A, GN=FLNA--96--G3
**V537**	SH3 domain-binding glutamic acid-rich-like protein 3, GN=SH3BGRL3--745--F18	**V36**	Filamin-A, GN=FLNA--97--H19
**V633**	SH3 domain-binding glutamic acid-rich-like protein 3, GN=SH3BGRL3--911--I19	**V247**	Fructose-bisphosphate aldolase A, GN=ALDOA--353--B14
**V239**	Stromal interaction molecule 2, GN=STIM2--344--A2	**V266**	Fructose-bisphosphate aldolase, GN=ALDOA--379--F14

**V530**	Thrombospondin-1, GN=THBS1--732--C6	**V42**	Gelsolin isoform 2, GN=GSN--104--I20
**V417**	Transcriptional repressor protein YY1, GN=YY1--568--E10	**V317**	Glutathione S-transferase omega-1, GN=GSTO1--441--I13
**V65**	Transketolase, GN=TKT--136--E6	**V403**	Glutathione--549--J5
**V611**	Tropomyosin 3, GN=TPM3--884--C12	**V56**	Heat shock cognate 71 kDa protein isoform 2, GN=HSPA8--
**V612**	Tropomyosin 3, GN=TPM3--885--C13	**V261**	Heterogeneous nuclear ribonucleoproteins A2/B1,
**V534**	Urea transporter 1, GN=SLC14A1--740--C7	**V63**	Keratin, type I cytoskeletal 10, GN=KRT10--131--G8
**V208**	Vimentin, GN=VIM--307--C18	**V118**	Keratin, type I cytoskeletal 10, GN=KRT10--199--F5
**V374**	Vimentin, GN=VIM--518--C21	**V75**	Keratin, type I cytoskeletal 9, GN=KRT9--148--H22
**V395**	Vimentin, GN=VIM--540--A6	**V301**	Keratin, type I cytoskeletal 9, GN=KRT9--423--B16
		**V24**	Keratin, type I cytoskeletal 9, GN=KRT9--81--F23
		**V210**	Leukocyte elastase inhibitor, GN=SERPINB1--309--I8
		**V407**	Mitochondrial carrier homolog 1 isoform 3, GN=MTCH1--554--H8
		**V361**	Myeloblastin, GN=PRTN3--503--B19
		**V91**	Myeloperoxidase, GN=MPO--168--B7
		**V466**	Peptidyl-prolyl cis-trans isomerase A, GN=PPIA--630--K6
		**V467**	Peptidyl-prolyl cis-trans isomerase A, GN=PPIA--632--I15
		**V347**	Peroxiredoxin-6, GN=PRDX6--481--H4
		**V202**	Phosphoglycerate kinase 1, GN=PGK1--300--J21
		**V29**	POTE ankyrin domain family member E, GN=POTEE—90-J9
		**V168**	POTE ankyrin domain family member F, GN=POTEF --261--101
		**V90**	Pyruvate kinase, GN=PKM--167--G10
		**V92**	Pyruvate kinase isozymes M1/M2, GN=PKM--169--J10
		**V334**	Ras suppressor protein 1, GN=RSU1--465--K4
		**V331**	Rho GTPase-activating protein 39, GN=ARHGAP39—462-K
		**V634**	Rho guanine nucleotide exchange factor 25, GN=ARHGEF25--
		**V73**	Serum albumin, GN=ALB--146--C15
		**V22**	Serum albumin, GN=ALB--79--F22
		**V398**	Superoxide dismutase, GN=SOD2--544--K14
		**V152**	Transcription factor 4, GN=TCF4--241--J1
		**V76**	Transcriptional repressor protein YY1, GN=YY1--149--H23
		**V251**	Tropomyosin 1 (Alpha) isoform 7, GN=TPM1--358--G24
		**V223**	Tropomyosin beta chain isoform 2, GN=TPM2--324--K24
		**V184**	UPF0515 protein C19orf66, GN=C19orf66--279--I6
		**V137**	UTP glucose-1-phosphate uridylyl transferase isoform 2, GN=UGP2--224--F9
		**V206**	Vimentin, GN=VIM--304--B11
		**V213**	Vimentin, GN=VIM--312--B12
		**V6**	Vinculin isoform 1, GN=VCL--57--H15
		**V7**	Vinculin isoform 1, GN=VCL--58--F20
		**V28**	Vinculin isoform 1, GN=VCL--89--K15
		**V3**	Vinculin isoform 1, GN=VCL--54--H14
		**V2**	Vinculin, GN=VCL--52--F1
		**V8**	Vinculin, GN=VCL--59--F21

**Clinically symptomatic (C/S)**		**Non-infected Healthy (N/H)**	
**V4**	Actin-related protein 2/3 complex subunit 3, GN=ARPC3--607-	**V1**	60 kDa heat shock protein, mitochondrial, GN=HSPD1--257--I3
**V6**	Actin, alpha 1, skeletal muscle, GN=ACTA1--890--E3	**V5**	Actin, alpha 1, skeletal, GN=ACTA1--121--I21
**V4**	Actin, cytoplasmic 1, GN=ACTB --640--13	**V3**	Actin, cytoplasmic 1, GN=ACTB--101--K16
**V2**	Actin, cytoplasmic 1, GN=ACTB--321--D5	**V8**	Actin, cytoplasmic 1, GN=ACTB--154--H24
**V2**	Actin, cytoplasmic 1, GN=ACTB--335--D6	**V9**	Actin, cytoplasmic 1, GN=ACTB--180--J11
**V2**	Actin, cytoplasmic 1, GN=ACTB--337--D7	**V1**	Actin, cytoplasmic 1, GN=ACTB--184--J12
**V3**	Actin, cytoplasmic 1, GN=ACTB--476--D10	**V1**	Actin, cytoplasmic 1, GN=ACTB--232--G15
**V3**	Actin, cytoplasmic 1, GN=ACTB--479--D11	**V1**	Actin, cytoplasmic 1, GN=ACTB--238--J16
**V4**	Actin, cytoplasmic 1, GN=ACTB--621--A11	**V1**	Actin, cytoplasmic 1, GN=ACTB--244--G18
**V4**	Actin, cytoplasmic 1, GN=ACTB--629--A13	**V1**	Actin, cytoplasmic 1, GN=ACTB--254--J17
**V4**	Actin, cytoplasmic 1, GN=ACTB--642--E15	**V1**	Actin, cytoplasmic 1, GN=ACTB--268--J19
**V4**	Actin, cytoplasmic 1, GN=ACTB--657--A14	**V1**	Actin, cytoplasmic 1, GN=ACTB--275--G22
**V4**	Actin, cytoplasmic 1, GN=ACTB--662--A16	**V2**	Actin, cytoplasmic 1, GN=ACTB--303--B10
**V4**	Actin, cytoplasmic 1, GN=ACTB--665--A17	**V1**	Actin, cytoplasmic 1, GN=ACTB--69--H16
**V5**	Actin, cytoplasmic 1, GN=ACTB--675--A18	**V5**	Actin, cytoplasmic 1, GN=ACTB--756--K8
**V5**	Actin, cytoplasmic 1, GN=ACTB--676--A19	**V5**	Actin, cytoplasmic 1, GN=ACTB--781--E1
**V5**	Actin, cytoplasmic 1, GN=ACTB--677--A20	**V1**	Actin, cytoplasmic 2, GN=ACTG1--255--I2
**V5**	Actin, cytoplasmic 1, GN=ACTB--679--A21	**V1**	Actin, cytoplasmic 2, GN=ACTG1--269--J2
**V5**	Actin, cytoplasmic 1, GN=ACTB--763--A24	**V5**	Actin, cytoplasmic 2, GN=ACTG1--118--K17
**V5**	Actin, cytoplasmic 1, GN=ACTB--769--B1	**V1**	Alpha-enolase, GN=ENO1--233--F10
**V6**	Actin, cytoplasmic 1, GN=ACTB--896--B5	**V1**	Alpha-enolase, GN=ENO1--234--J15
**V6**	Actin, cytoplasmic 1, GN=ACTB--897--B6	**V1**	Alpha-enolase, GN=ENO1--240--G17
**V6**	Actin, cytoplasmic 2, GN=ACTG1--891--E4	**V1**	Alpha-enolase, GN=ENO1--247--G19
**V4**	Actin, cytoplasmic 2, GN=ACTG1--610--D16	**V1**	Alpha-enolase, GN=ENO1--256--G20

**V4**	Actin, cytoplasmic 2, GN=ACTG1--613--D17
**V4**	Actin, cytoplasmic 2, GN=ACTG1--623--A12
**V4**	Actin, cytoplasmic 2, GN=ACTG1--659--A15
**V5**	Actin, cytoplasmic 2, GN=ACTG1--759--D22
**V5**	Actin, cytoplasmic 2, GN=ACTG1--762--L14
**V4**	Alpha-actinin-1, GN=ACTN1--588--A8
**V4**	Annexin, GN=ANXA1--627--3
**V5**	ATP synthase subunit alpha, mitochondria, GN=ATP5A1--737-
**V4**	Bestrophin-3, GN=BEST3--671--B23
**V5**	Centromere protein H, GN=CENPH--686--D2
**V4**	Ferritin light chain --572--20
**V6**	Fibrinogen alpha chain isoform 2, GN=FGA--889--H12
**V5**	Fibrinogen alpha chain isoform 2, GN=FGA--127--G7
**V5**	Hemoglobin subunit beta, GN=HBB--685--L12
**V5**	Hemoglobin subunit beta, GN=HBB--690--L13
**V6**	Hemoglobin subunit beta, GN=HBB--904--L15
**V6**	Hemoglobin subunit beta, GN=HBB--905--K23
**V4**	Heterogeneous nuclear ribonucleoprotein K, GN=HNRNPK--
**V5**	Histone H4, GN=HIST1H4A--683--A22
**V5**	Histone H4, GN=HIST1H4A--696--A23
**V5**	Histone H4, GN=HIST1H4A--725--E18
**V5**	Histone H4, GN=HIST1H4A--772--B2
**V5**	Keratin, type I cytoskeletal 9, GN=KRT9--863--E2
**V5**	Keratin, type II cuticular Hb3, GN=KRT83--797--E20
**V4**	Keratin, type II cytoskeletal 1, GN=KRT1--582--A7
**V5**	Keratin, type II cytoskeletal 1, GN=KRT1--738--D21
**V5**	Keratin, type II cytoskeletal 1, GN=KRT1--761--D23
**V5**	Keratin, type II cytoskeletal 1, GN=KRT1--796--E19
**V5**	LVV-hemorphin-7, GN=HBB--867--C11
**V4**	Mitochondrial carrier homolog 1 isoform 3, GN=MTCH1--552-
**V4**	Mitochondrial carrier homolog 1 isoform 3, GN=MTCH1--583-
**V4**	Mitochondrial carrier homolog 1 isoform 3, GN=MTCH1--620-
**V4**<br>**93**	Mitochondrial carrier homolog 1 isoform 3, GN=MTCH1--666-<br>-B22
**V5**	Mitochondrial carrier homolog 1 isoform 3, GN=MTCH1--707-
**V4**	Myosin light polypeptide 6, GN=MYL6--628--E14
**V4**	Myosin regulatory light polypeptide 9, GN=MYL9--612--K21
**V5**	Myotrophin, GN=MTPN--712--I17
**V5**	Protein S100-A6, GN=S100A6--721--C5
**V5**	Protein S100-A6, GN=S100A6--866--C10
**V5**	Ras-related protein Ral-B, GN=RALB--718--C4
**V5**	Ras-related protein Ral-B, GN=RALB--787--K9
**V5**	Ras-related protein Rap-1b --744--23
**V5**<br>**18**	SH3 domain-binding glutamic acid-rich-like protein 2,<br>GN=SH3BGRL2--704--I16
**V5**<br>**37**	SH3 domain-binding glutamic acid-rich-like protein 3,<br>GN=SH3BGRL3--745--F18
**V6**	SH3 domain-binding glutamic acid-rich-like protein 3,
**V2**	Stromal interaction molecule 2, GN=STIM2--344--A2
**V5**	Thrombospondin-1, GN=THBS1--732--C6
**V4**	Transcriptional repressor protein YY1, GN=YY1--568--E10
**V6**	Transketolase, GN=TKT--136--E6
**V5**	Urea transporter 1, GN=SLC14A1--740--C7
**V3**	Vimentin, GN=VIM--540--A6

**V2**	Annexin A1, GN=ANXA1--373--H1
**V1**	ATP synthase subunit beta, GN=ATP5B--219--G14
**V4**	Cofilin 1 (Non-muscle), GN=CFL1--611--K5
**V2**	Cyclin-dependent kinase 4, GN=CDK4--330--I11
**V1**	Elongation factor Tu, mitochondrial, GN=TUFM--260--I4
**V1**	Elongation factor Tu, mitochondrial, GN=TUFM--262--I5
**V1**	Fibrinogen alpha chain isoform 2--273--104
**V1**	Fibrinogen alpha chain isoform 2, GN=FGA--208--I24
**V1**	Fibrinogen alpha chain isoform 2, GN=FGA--271--G21
**V1**	Fibrinogen beta chain, GN=FGB--195--G11
**V1**	Fibrinogen beta chain, GN=FGB--197--G12
**V1**	Fibrinogen beta chain, GN=FGB--200--I22
**V1**	Fibrinogen beta chain, GN=FGB--202--I23
**V1**	Fibrinogen beta chain, GN=FGB--205--C16
**V1**	Fibrinogen beta chain, GN=FGB--214--C17
**V3**	Filamin-A, GN=FLNA--100--H20
**V4**	Filamin-A, GN=FLNA--102--G5
**V4**	Filamin-A, GN=FLNA--103--H21
**V3**	Filamin-A, GN=FLNA--91--H17
**V3**	Filamin-A, GN=FLNA--92--F24
**V3**	Filamin-A, GN=FLNA--93--H18
**V3**	Filamin-A, GN=FLNA--94--G1
**V3**	Filamin-A, GN=FLNA--95--G2
**V3**	Filamin-A, GN=FLNA--96--G3
**V3**	Filamin-A, GN=FLNA--97--H19
**V2**	Fructose-bisphosphate aldolase A, GN=ALDOA--353--B14
**V2**	Fructose-bisphosphate aldolase, GN=ALDOA--379--F14
**V4**	Gelsolin isoform 2, GN=GSN--104--I20
**V3**	Glutathione S-transferase omega-1, GN=GSTO1--441--I13
**V4**	Glutathione--549--J5
**V2**	Glyceraldehyde-3-phosphate dehydrogenase, GN=GAPDH--346--
**V2**	Glyceraldehyde-3-phosphate dehydrogenase, GN=GAPDH--367--F13
**V3**<br>**07**	Haloacid dehalogenase-like hydrolase domain-containing protein 2,<br>GN=HDHD2--431--H3
**V5**	Heat shock cognate 71 kDa protein isoform 2, GN=HSPA8--124--G6
**V2**	Heterogeneous nuclear ribonucleoproteins A2/B1,
**V6**	Keratin, type I cytoskeletal 10, GN=KRT10--131--G8
**V1**	Keratin, type I cytoskeletal 10, GN=KRT10--199--F5
**V7**	Keratin, type I cytoskeletal 9, GN=KRT9--148--H22
**V3**	Keratin, type I cytoskeletal 9, GN=KRT9--423--B16
**V2**	Keratin, type I cytoskeletal 9, GN=KRT9--81--F23
**V3**	Keratin, type II cytoskeletal 1 --502--8
**V3**	Keratin, type II cytoskeletal 1, GN=KRT1--533--D15
**V1**<br>**54**	Lactotransferrin isoform delta, GN=LTF--243--B8
**V4**<br>**07**	Mitochondrial carrier homolog 1 isoform 3, GN=MTCH1--554--H8
**V3**	Myeloblastin, GN=PRTN3--503--B19
**V1**	Myeloperoxidase isoform H14, GN=MPO--272--B9
**V9**	Myeloperoxidase, GN=MPO--168--B7
**V4**	Peptidyl-prolyl cis-trans isomerase A, GN=PPIA--630--K6
**V5**	Peptidyl-prolyl cis-trans isomerase A, GN=PPIA--632--I15
**V3**	Peroxiredoxin-6, GN=PRDX6--481--H4
**V2**	Phosphoglycerate kinase 1, GN=PGK1--300--J21
**V2**	Phosphoglycerate kinase 1, GN=PGK1--305--D4
**V2**	POTE ankyrin domain family member E, GN=POTEE--90--J9
**V3**	Proteasome subunit beta type-3, GN=PSMB3--531--J4
**V2**	Purine nucleoside phosphorylase, GN=PNP--403--J24
**V9**	Pyruvate kinase isozymes M1/M2, GN=PKM--169--J10
**V9**	Pyruvate kinase, GN=PKM--167--G10
**V3**	Ras suppressor protein 1, GN=RSU1--465--K4
**V3**	Rho GTPase-activating protein 39, GN=ARHGAP39--462--K3
**V6**<br>**34**	Rho guanine nucleotide exchange factor 25, GN=ARHGEF25--913--<br>J8
**V2**	Serum albumin, GN=ALB--79--F22
**V3**	Superoxide dismutase, GN=SOD2--544--K14
**V1**	Transcription factor 4, GN=TCF4--241--J1
**V2**	Tropomyosin 1 (Alpha), GN=TPM1--358--G24
**V2**	Tropomyosin beta chain isoform 2, GN=TPM2--324--K24
**V1**	UPF0515 protein C19orf66, GN=C19orf66--279--I6
**V1**<br>**37**	UTP glucose-1-phosphate uridylyl transferase isoform 2, GN=UGP2--<br>224--F9

	**V2**	Vimentin, GN=VIM--304--B11
	**V2**	Vimentin, GN=VIM--312--B12
	**V3**	Vinculin isoform 1, GN=VCL--54--H14
	**V6**	Vinculin isoform 1, GN=VCL--57--H15
	**V7**	Vinculin isoform 1, GN=VCL--58--F20
	**V2**	Vinculin isoform, GN=VCL--89--K15
	**V2**	Vinculin, GN=VCL--52--F1
	**V8**	Vinculin, GN=VCL--59--F21

Proteome datasets from N/H, n=30; C/A, n=25; C/S, n=28 subjects were subjected to bipartite network analysis as described in Materials and Methods. Of the 635 protein spots analyzed, 194 and 208 spots were univariably significant in C/A and C/S groups, respectively (vs N/H controls, p<0.001) after false discovery rate (FDR) correction. Note that some proteins, e.g., ACTB had specific peptides that were differentially expressed in compared groups.

Table S2: Summary of Receiver Operating Characteristic (ROC) analysis of secreted biomarkers.

Independent Variable	C/A + C/S vs N/H			C/A vs C/S			C/S vs C/A		
	AUC	TPR	FPR	AUC	TPR	FPR	AUC	TPR	FPR
hnRNPA1	**0.986**	0.950	0.875	0.654	0.733	0.367	0.346	0.433	0.667
Vimentin	**0.953**	0.783	0.868	0.066	0.167	0.833	**0.934**	**0.960**	0.233
PARP1	**0.999**	0.992	0.962	0.441	0.467	0.633	0.559	0.567	0.500
8-OHdG	**0.996**	0.967	0.958	0.074	0.067	0.867	**0.926**	**0.933**	0.167
Copeptin	**0.998**	0.983	0.959	0.009	0.033	0.967	**0.991**	**0.967**	0.200
Endostatin	**0.935**	0.633	0.956	0.103	0.067	0.933	0.897	**0.900**	0.267
Myostatin	0.277	0.167	0.967	**0.998**	0.967	0.033	0.002	0.067	**0.967**

Area under ROC curve (AUC) analyses was performed to calculate the performance of independent variables across classification thresholds. AUC results were interpreted as no discrimination (0–0.5), average (0.5–0.7), acceptable (0.7–0.8), good (0.8–0.9), or excellent (0.9–1.0) performance of independent variables in distinguishing the infection or disease state. True positive rate (TPR=Sensitivity) and False positive rate (FPR=1-Specificity) values exhibit the ability of the test to correctly identify disease group, higher the values better the model is performing.

Table S3. Demographics of subjects enrolled for biomarkers analyses.

Clinical characterization	Subjects	Age in years (mean ± SD)	Sex males (%)
Seropositive, clinically asymptomatic (C/A)	n = 30	49.8 ± 9.2	14 (46.6%)
Seropositive, clinically symptomatic (C/S)	n = 30	53.1 ± 10.6	16 (53.3%)
Seronegative, non-infected, healthy (N/H)	n = 24	39 ± 16.2	15 (50%)

Subjects were screened for *T. cruzi*-specific antibodies by Wiener Chagatest-ELISA and Wiener Chagastest-HAI kits. Clinical exam included physical exam, electrocardiography, and echocardiography. Seropositive individuals with none-to-minor echocardiography abnormalities, no left ventricular dilatations, preserved systolic function (ejection fraction: 55-70%) were considered clinically asymptomatic. Seropositive individuals with varying degree of heart involvement evidenced by systolic dysfunction (ejection fraction: < 55%), left ventricular dilatation (diastolic diameter ≥ 57 mm), and/or potential signs of congestive heart failure were classified as clinically symptomatic.

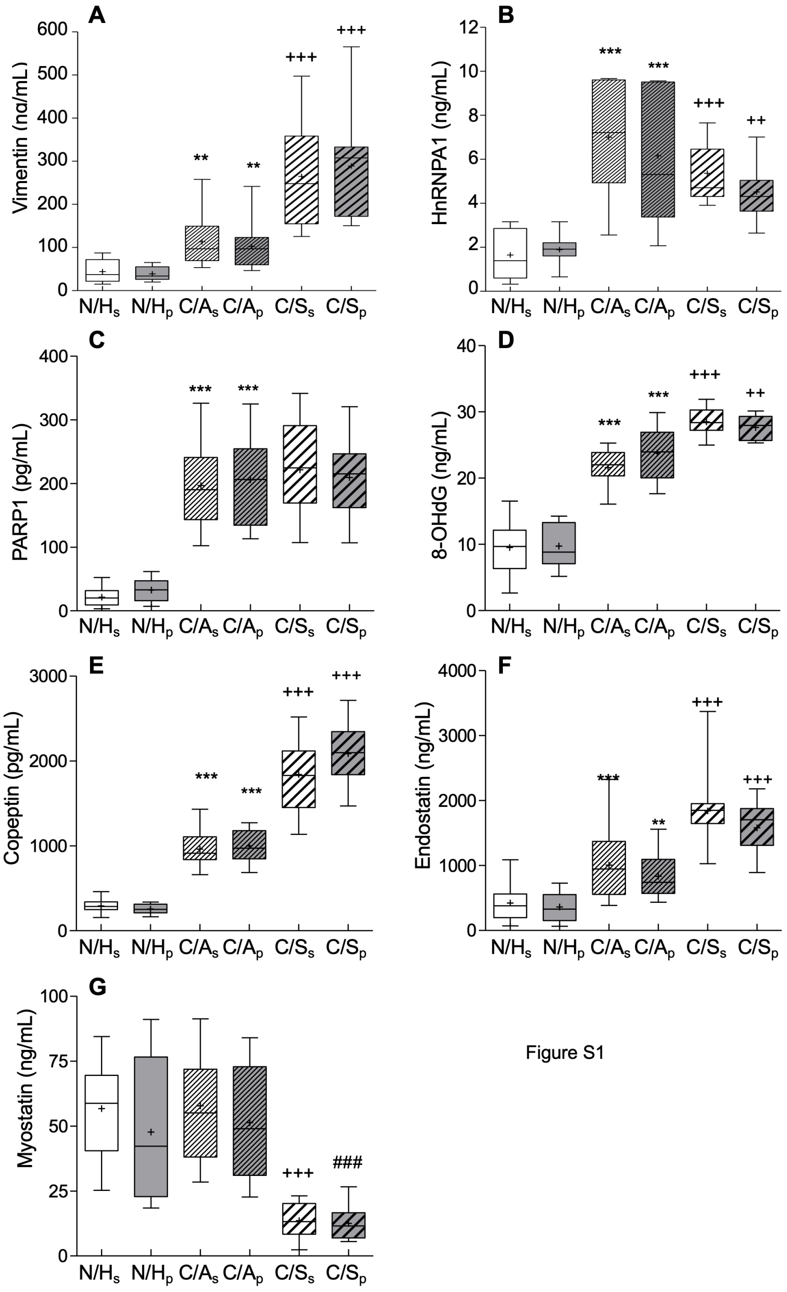

Figure S1

Figure S1. Blood biomarkers are equally presented in serum or plasma. Box plots show serum (s) or plasma (p) levels of **(A)** vimentin, **(B)** hnRNPA1, **(C)** PARP1, **(D)** 8-OHdG, **(E)** copeptin, **(F)** endostatin, and **(G)** myostatin determined by an ELISA. The horizontal lines of the box (bottom to top) depict the lower quartile (Q1; lowest 25%), median (Q2; middle value), and upper quartile (Q3; highest 25%). The lower and upper whiskers depict the smallest and largest nonoutlier observations, respectively. The spacing between the different parts of the box indicates the degree of dispersion. Significance is presented as *N/H vs C/A or C/S (Student's two tailed t test) and $^{+}$ C/A vs C/S (one way ANOVA followed by Tukey's post hoc correction test) and annotated with one ($p \leq 0.05$), two ($p \leq 0.01$), or three ($p \leq 0.001$) symbols.

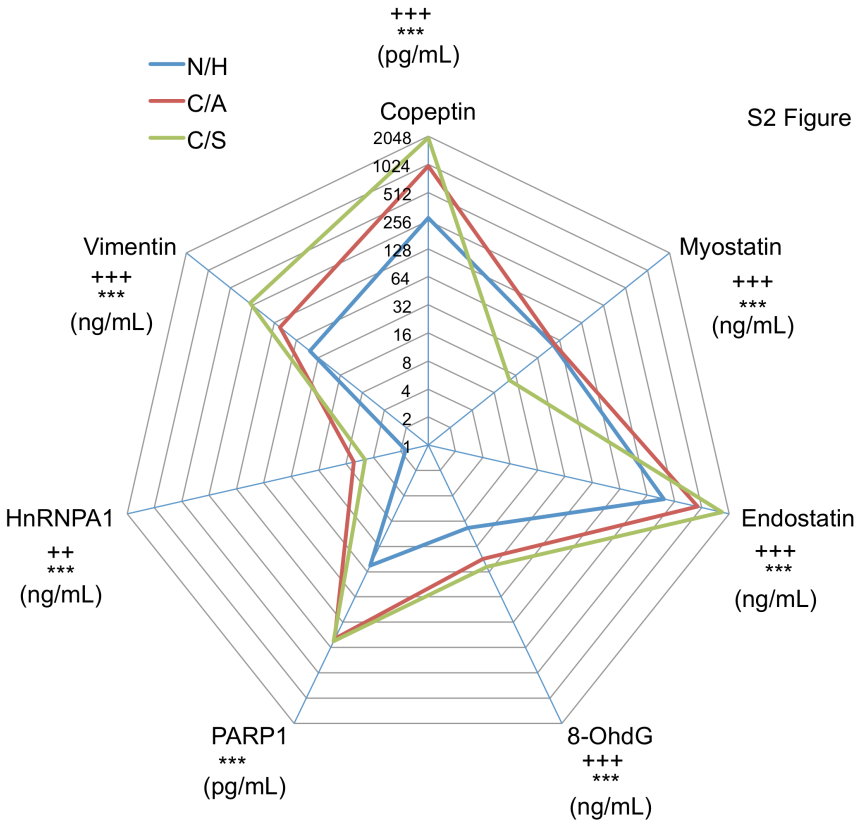

Figure S2. Radar plot displaying all biomarkers data across the studied groups. Shown are mean values for secreted levels of hnRNPA1, vimentin, PARP1, 8-OHdG, copeptin, endostatin and myostatin in enrolled studied groups (N/H, C/A, C/S). Mean values were fitted in log scale with base value of 2. Each parameter with its own axis and common central point has a minimum range starting from 1 to maximum range 2048. Significance is presented as * (N/H vs C/A + C/S) or $^{+}$ (C/A vs C/S), and annotated with one (p≤0.05), two (p≤0.01), or three (p≤0.001) symbols.
